# Psychological Status and Quality of Life in relation to the Metabolic Syndrome: Isfahan Cohort Study

**DOI:** 10.1155/2012/380902

**Published:** 2012-05-20

**Authors:** Hamidreza Roohafza, Masoumeh Sadeghi, Mohammad Talaei, Zahra Pourmoghaddas, Nizal Sarrafzadegan

**Affiliations:** ^1^Cardiovascular Research Center, Isfahan Cardiovascular Research Institute (WHO-Collaborating Center), Isfahan University of Medical Sciences, 81465-1148 Isfahan, Iran; ^2^Cardiac Rehabilitation Research Center, Isfahan Cardiovascular Research Institute (WHO-Collaborating Center), Isfahan University of Medical Sciences, P.O. Box 81465-1148, Isfahan, Iran; ^3^Child Health Promotion Research Center, School of Medicine, Isfahan University of Medical Sciences, 81465-1148 Isfahan, Iran

## Abstract

*Objective*. Current study was designed to investigate the association of metabolic syndrome (MetS) with depression, anxiety, psychological distress, and quality of life (QoL). *Design*. Two hundred and fifteen contributors with MetS and 253 participants without MetS were randomly selected from 2151 participants of Isfahan Cohort Study who were residents of Isfahan city. Measurements consisted of fasting blood samples, anthropometrics, and self-reported data of 12-item General Health Questionnaire, Hospital Anxiety and Depression Scale, and European Quality of Life-5 Dimension. Binary logistic regression analysis was used to find the association between MetS and four psychological factors. *Results*. Participants mean age was 56.3 ± 9.8 years. Male/female ratio was 0.86 (217/251). Mean score of depression (*P* = 0.003), anxiety (*P* = 0.018), distress (*P* = 0.047), and QoL (*P* ≤ 0.001) was significantly higher in MetS group. There were significant increasing relationships between depression (OR 1.10, 95% CI 1.03–1.22), anxiety (OR 1.03, 95% CI 1.05–1.11), and QoL (OR 1.13, 95% CI 1.05–1.23) and MetS when associations were adjusted for other risk factors, but it was not the case for distress (OR 1.03, 95% CI 0.99–1.08). *Conclusion*. It might be better to consider MetS as a combination of biological and psychological risk factors. Thus, a person with metabolic disease should be recognized as a patient with these factors and be screened for all of them.

## 1. Introduction

Assuming that psychological status affects all medical conditions, researchers have focused on these psychological status' roles especially on noncommunicable disease with almost unknown origin [1]. The relationship of depression, anxiety, and psychological distress with chronic metabolic disease such as diabetes mellitus, insulin resistance, and dyslipidemia has been established [2]. Metabolic syndrome (MetS) is a combination of metabolic risk factors such as impaired glucose level, dyslipidemia, hypertension, and central obesity that predispose persons to cardiovascular mortality and morbidity [3]. Obvious causes for this chronic condition have not been introduced and contributory factors in MetS have been receiving attention [4].

There are conflicting results about probable association of MetS with psychological problems. For instance, it was shown that MetS associated with long-term depressive symptoms [5, 6], while another study reported absence of any association between MetS, depression and anxiety [7]. Other lines of evidence have introduced prolong exposure to chronic psychological distress as an important risk factor for MetS and have claimed there is an association between daily life stressors and metabolic syndrome [8]; however, empirical data on this field is little and needs more evaluation. On the other hand, the relationship of lower physical well-being and social function as indicators of QoL with MetS has been shown [9]; however, a study ascribed this association to obesity rather than MetS [10]. In general, findings about association of MetS and poor quality of life (QoL) are scarce.

MetS prevalence in Iranian population comparing with other Asian societies is high, varied from 11% of young adolescents under twenty years of age to 33% of persons who aged more [11, 12]. The global belief of this high prevalence is socioeconomic and demographics changes [13]. Similarly, the number of Iranian patients who suffer from anxiety, depression, and psychological distress is high [14]. Therefore, we aimed to investigate association of depression, anxiety, psychological distress, and quality of life with metabolic syndrome in a sample of Iranian as a population with high prevalence of MetS.

## 2. Methods and Materials

This cross-sectional study was designed among participants of Isfahan Cohort Study (ICS). ICS is a population-based cohort study on 6504 participants who were ≥35 years old from three counties of central Iran (Isfahan, Arak, Najafabad) [15, 16], which started in 2001, for detecting mortality, morbidity, and risk factors of cardiovascular disease. Approvals were made with the bioethical committee of the Provincial University of Medical Sciences. Informed consents were obtained from participants of both groups.

There were 1520 subjects available in Isfahan city from which 599 participants met Adult Treatment Panel III (ATPIII) criteria for detecting Mets at baseline that was described in details elsewhere [17]. It was calculated that 254 subjects in each group would be required to detect a difference of at least 1 (effect size) in scores of psychological factors between MetS and non-MetS groups with 80% power (*β* = 0.2) at the 2-tailed 0.025 level of significance (*α* = 0.05) and standard deviation of 4 in both groups. Using random sampling procedure in SPSS, 254 individuals with MetS and the same number without it were selected and invited for filling questionnaires and new anthropometric and biochemical measurements. The response rate in MetS group was lower than non-MetS group (86.6% versus 96.4%); therefore, the sample size of control group was increased to 300 subjects using this equation: 1/*n*_case_ + 1/*n*_control_ = 2/*n*_calculation_. According to the new measurements, 5 participants did not fulfill ATPIII criteria for MetS anymore. In turn, 47 subjects without history of MetS based on baseline measurements met MetS criteria based on new measurements. In order to evaluate persistent MetS, these individuals were excluded and 215 subjects with MetS and 253 without it were included in analysis. Power analysis was carried out using G*Power 3.1.3 (18) for the GHQ12 scores as the factor with the weakest association in this study (Table 2). Considering the actual sample size and real findings on this score, the minimum power of study was calculated as 81.7% (post hoc power analysis).

### 2.1. Measurements

Body weight and height were measured with indoor clothing and light slipper. Waist circumference (WC) was measured by tape, horizontally 1 cm above the navel. Blood pressure was taken two times after five minutes resting in calm and comfortable room with 15-minutes interval; finally mean of two blood pressure recordings was used for analysis. Triglyceride (TG), fasting plasma glucose (FPG), and high-density lipoprotein cholesterol (HDL-C) were detected by an enzymatic method in Elan 2000 autoanalyzer. Friedewald formula was used for calculating low-density lipoprotein cholesterol (LDL-C) level except in individuals with TG ≥ 400 mg/dL which LDL-C level was measured directly. Participants who used at least one cigarette per day were considered as current smokers.

Self-administered 12-item General Health Questionnaire (GHQ12) was used for assessing distress [18, 19]. Less than usual, no more than usual, fairly more than usual, or much more than usual were answers to questions of this questionnaire, which were scaled as GHQ score method (0-0-1-1 method). According to this method sum of answers to total items were scored on a scale ranging from 0 to 12. Participants with higher score was considered as higher psychological distress.

Anxiety and depression were evaluated by the Hospital Anxiety and Depression Scale (HADS) [20, 21]. The HADS is a 14-item questionnaire consisting of two anxiety and depression subscales; each one includes seven items. Four point scale used for rating anxiety and depression, ranged from 0 to 21 with a cut-off value of 7. In each subscales, higher score showed higher anxiety and depression.

Self-administered instrument European Quality of Life-5 Dimensions (EQ-5D) was used to evaluate QoL [22]. Five dimensions of health including mobility, self-care, usual activity, pain/discomfort and anxiety/depression were evaluated by this instrument. Three levels of severity presented for each domain as 1 (No problems), 2 (some problems), and 3 (extreme problems). Global QoL score of participants was defined by the combinations of dimensions' score. Higher EQ-5D scores indicate poor QoL.

### 2.2. Statistical Analysis

Data entry was done using Epi Info, Version 6 (Centers for Disease Control, Atlanta, GA). Data were analyzed by SPSS software, version 15 (SPSS Inc, Chicago, IL). A *P* value ≤ 0.05 was considered statistically significant for all analyses. Student's *t*-test for continuous variables and chi-square test for discrete variables were used.

Binary logistic regression analysis was used to find the association among the MetS and four psychological variables. Odds ratios (OR) were reported with the corresponding 95% confidence intervals. Independent variables included scores of depression, anxiety, distress, and QoL, as well as smoking, body mass index (BMI), total cholesterol, age, sex, and education. Dependent variable was MetS. Linear regression analyses were used to evaluate the relationship of each psychological variable with the number of MetS components as dependent variable.

## 3. Results

The mean age of all participants in present study was 56.3 ± 9.8 years. Male/female ratio was 0.86 (217/251) (Table 1). In non-MetS group, 55 (21.7%), 95 (37.5%), and 103 (40.8%) participants had 0, 1, and 2 components of MetS, respectively. In MetS group, 133 (61.8%), 66 (30.7%) and 16 (7.4%) individuals were detected that had 3, 4, and 5 component, respectively.

Seventy persons (32.5%) in MetS group and 7 (2.7%) individuals in non-MetS group had FPG ≥ 110 mg/dL (*P* < 0.001). One hundred and ninety three (89.7%) subjects in MetS group and 86 (33.9%) in non-MetS group had TG ≥ 150 mg/dL (*P* < 0.001). In MetS group, 171 (79.5%) individuals had low HDL-C level (<40 mg/dL for men and <50 mg/dL for women), but it was detected only in 86 (33.9%) subjects in non-MetS group (*P* < 0.001). High WC (≥102 cm in men and ≥88 cm in women) was detected in 167 (77.6%) and 48 (19%) participants in MetS and non-MetS group, respectively (*P* < 0.001). Likewise, 143 (66.5%) and 81 (32%) participants in these groups had high blood pressure (systolic blood pressure ≥130 or diastolic blood pressure ≥85), respectively (*P* < 0.001).

Mean score of depression, anxiety, distress, and Qol was significantly higher in MetS group as were represented in Table 2. As shown in Table 3, the multivariate logistic regression analysis after adjusting for demographic risk factors, showed the significant association of depression, anxiety, distress, and quality of life score with MetS. By adjusting age, sex, smoking, BMI, and cholesterol, distress role attenuated but depression, anxiety, and QoL role significantly remained in the model.

**Figure 1:**
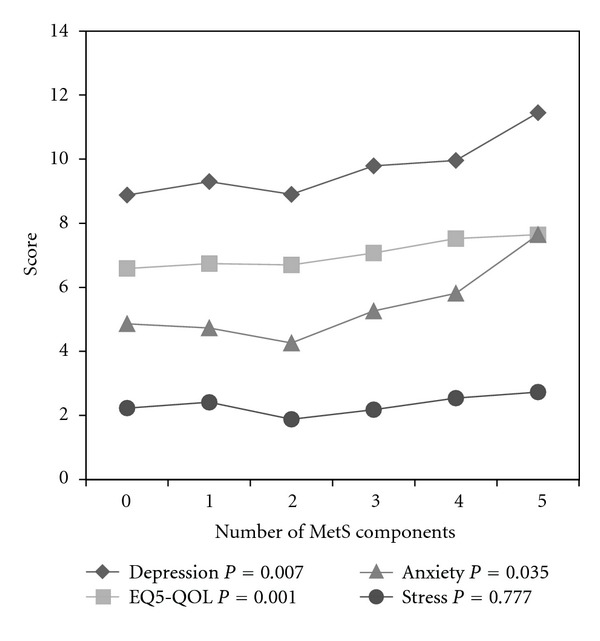
Psychological variable according to the number of metabolic syndrome components.

Figure 1 illustrates significant worsening trends of scores for depression, QoL, and anxiety as the number of MetS components increased; however, distress scores did not show this pattern. Among components of MetS, central obesity was significantly associated with detrimental increase of all four psychological factors (*P* < 0.001 except for GHQ-12 that *P* = 0.03). Depression score and QoL were significantly lower in high FPG (*P* = 0.002, *P* = 0.008). Conversely, GHQ-12 score was slightly lower in those without TG component of MetS (*P* = 0.037). There were rare reports of drugs that have effect on mood (*n* = 15, including 8 for TCAs, 4 for sodium valproate, and 6 for SSRIs, some reported concurrent use of 2 drugs).

## 4. Discussion

Present study participants with clinically verified MetS had a significant higher score for depression, anxiety, and distress. This study showed individuals with MetS had impaired QoL.

Current study suggested the association between MetS and depression. In line with our findings, a community-based study [23] found that MetS is associated with self-perceived depression. In a cohort study [24], MetS could predict depression. While another research [5] on depressed patients indicated the association of long-term depressive symptoms and emergence of MetS, Räikköonen et al. [6] have shown in their study that psychological factors play causal role in MetS. Hence, it seems there is a two-way street between depression and MetS. 

In spite of different study methods, our results are in agreement with Räikkönen et al. [25] and Carroll et al. [26] and showed that anxiety is associated with MetS in both sexes. Other findings of Räikkönen's studies demonstrated that feeling intense, anger, and distress increased risk for MetS [6, 25]. Furthermore, other researchers [8] have suggested emergence of MetS after 5 years exposure to high psychological distress. Statistically significant association of our participants' distress with MetS was lost after adjusting for BMI, smoking, age, sex, and cholesterol level. Different measurement methods of psychological distress, type of research, study population, and adjusting factors in present study compared to Räikkönen et al. researches may justify these differences.

Our findings showed that individuals with MetS had impaired QoL in terms of health, mobility, self-care, usual activity, pain/discomfort, and anxiety/depression, even after controlling confounding factors. Two different studies [9, 13] have demonstrated that people with MetS have impaired QoL including physical and social function as well as general and mental health. Although studies about QoL and MetS are few, but present study findings were in accordance with them and have suggested impaired quality of life in patients with MetS.

Some mechanisms were suggested as possibly responsible for association of psychological disorders, impaired QoL and MetS. First mechanism is biological alternation [27] such as autonomic nervous system changes like heart rate variability, dysregulation of endocrine organ like hypothalamic-pituitary-adrenal axis, and alternation of inflammatory and hemostatic markers and neurotransmitters especially blunted level of serotonin [6]. Second mechanism is separated correlation of psychological problems with MetS components, for instance, association of depression with visceral fat accumulation, insulin resistance, and dyslipidemia [2]. Last one is the similar risk factors of psychological, and metabolic disorders [9], for example, low intensity of leisure time physical activity, unhealthy dietary habits, high consumption of alcohol, and low education, and so forth.

This study benefited from participants with wide range of age, enough sample size, and recruiting individuals who had MetS for about 7 years. However, some limitations should be considered. First, self-reported questionnaires such as HADS give limited data about participants' depression and anxiety at one time but no information about lifetime psychological problems. Second, in spite of wide level of adjustment in analyses of present study, it is still possible that unmeasured confounders interfere with part of the association between MetS and psychological disorders. Third, recall bias may occur because participants with MetS may report poorer social and emotional situation because of the syndrome. In addition, cross-sectional design of study makes temporality problem that causes difficulties in interpretation of cause and effect.

## 5. Conclusion

MetS in addition to biological causes might be a combination of psychological problem such as depression, anxiety, psychological distress, and impaired Qol. Thus, a person with metabolic disease should be recognized as a patient with biological, psychological, and environmental risk factors and screened for all of them.

## Figures and Tables

**Table 1:** Baseline characteristics according to the presence or absence of metabolic syndrome.

	Non-MetS (*n* = 253)	MetS (*n* = 215)	*P*-value
	Mean ± SD	Mean ± SD	
Age	56.3 ± 9.5	56.5 ± 9.4	0.791
Male, *n* (%)	148 (58.4%)	69 (32.1%)	<0.001
Current smoking, *n* (%)	41 (16.2%)	21 (9.7%)	<0.001
Fasting plasma glucose (mg/dL)	84.0 ± 19.7	113.3 ± 48.1	<0.001
Waist circumference (cm)	89.1 ± 9.6	99.9 ± 9.1	<0.001
Body mass index (kg/m^2^)	27.1 ± 4.1	31.5 ± 4.9	<0.001
Systolic blood pressure (mm/Hg)	120.8 ± 18.5	131.0 ± 16.8	<0.001
Diastolic blood pressure (mm/Hg)	77.8 ± 8.1	81.8 ± 9.1	<0.001
Low-density lipoprotein cholesterol (mg/dL)	118.6 ± 25.2	120.4 ± 27.8	0.463
High-density lipoprotein cholesterol (mg/dL)	48.4 ± 11.6	41.6 ± 9.3	<0.001
Total cholesterol (mg/dL)	206.3 ± 38.7	217.3 ± 45.2	0.007
Triglycerides (mg/dL)	144.5 ± 68.7	263.9 ± 67.4	<0.001

**Table 2:** Psychological variable according to the presence or absence of metabolic syndrome.

	Non-MetS (*n* = 253)	MetS (*n* = 215)	*P*-value
	Mean ± SD	Mean ± SD	
Depression score	9.04 ± 3.09	9.96 ± 3.32	0.003
Anxiety score	4.56 ± 4.27	5.59 ± 4.77	0.018
GHQ score	2.05 ± 1.52	2.42 ± 1.60	0.047
EQ5-QOLscore	6.69 ± 1.45	7.25 ± 1.47	<0.001

**Table 3:** Unadjusted and fully adjusted ORs of psychological Factors with metabolic syndrome.

	Unadjusted	Age and sex adjusted	Fully adjusted*
	OR (95% CI)	OR (95% CI)	OR (95% CI)
Depression score	1.23 (1.07–1.41)^‡^	1.17 (1.06–1.34)^‡^	1.10 (1.03–1.22)^†^
Anxiety score	1.12 (1.04–1.21)^†^	1.11 (1.05–1.19)^†^	1.03 (1.05–1.11)^†^
GHQ score	1.08 (1.03–1.12)^†^	1.04 (1.02–1.18)^†^	1.03 (0.99–1.08)
EQ5-QOL score	1.47 (1.29–1.69)^§^	1.24 (1.07–1.44)^‡^	1.13 (1.05–1.23)^†^

*Adjusted for age, sex, smoking, BMI and total cholesterol.

^†^ *P* < 0.05, ^‡^ *P* < 0.01, ^§^ *P* < 0.001.
